# Identification and Computational Analysis of Rare Variants of Known Hearing Loss Genes Present in Five Deaf Members of a Pakistani Kindred

**DOI:** 10.3390/genes12121940

**Published:** 2021-11-30

**Authors:** Irum Badshah Saleem, Muhammad Shareef Masoud, Muhammad Qasim, Muhammad Ali, Zubair M. Ahmed

**Affiliations:** 1 Department of Bioinformatics and Biotechnology, Government College University, Faisalabad 38000, Pakistan; badshah.saleem85@gmail.com (I.B.S.); qasemawan@gmail.com (M.Q.); 2 Department of Animal Sciences, Quaid-e-Azam University, Islamabad 46000, Pakistan; vc@qau.edu.pk; 3 Department of Otorhinolaryngology Head and Neck Surgery, University of Maryland School of Medicine, Baltimore, MD 21201, USA

**Keywords:** hearing loss, whole exome sequencing, genetic heterogeneity, digenic, *KCNQ4*, *CDH23*, *SLC26A4*, *GJB2*, *MPDZ*

## Abstract

Hearing loss (HL) is the most common neurosensory defect in humans that affects the normal communication. Disease is clinically and genetically heterogeneous, rendering challenges for the molecular diagnosis of affected subjects. This study highlights the phenotypic and genetic complexity of inherited HL in a large consanguineous Pakistan kindred. Audiological evaluation of all affected individuals revealed varying degree of mild to profound sensorineural HL. Whole exome (WES) of four family members followed by Sanger sequencing revealed candidate disease-associated variants in five known deafness genes: *GJB2* (c.231G>A; p.(Trp77 *)), *SLC26A4* (c.1337A>G; p.(Gln446Arg)), *CDH23* (c.2789C>T; p.(Pro930Leu)), *KCNQ4* (c.1672G>A; p.(Val558Met)) and *MPDZ* (c.4124T>C; p.(Val1375Ala)). All identified variants replaced evolutionary conserved residues, were either absent or had low frequencies in the control databases. Our in silico and 3-Dimensional (3D) protein topology analyses support the damaging impact of identified variants on the encoded proteins. However, except for the previously established “pathogenic” and “likely pathogenic” categories for the c.231G>A (p.(Trp77 *)) allele of *GJB2* and c.1377A>G (p.(Gln446Arg)) of *SLC26A4,* respectively, all the remaining identified variants were classified as “uncertain significance” based on the American College of Medical Genetics and Genomics/Association for Molecular Pathology (ACMG/AMP) variant pathogenicity guidelines. Our study highlights the complexity of genetic traits in consanguineous families, and the need of combining the functional studies even with the comprehensive profiling of multiple family members to improve the genetic diagnosis in complex inbred families.

## 1. Introduction

Hearing loss (HL) is a genetic hereditary as well as clinically heterogeneity disorder. According to World Health Organization (WHO) HL is a widespread sensorineural defect (>25 dB in children and >40 dB in adults) [1], which influences around 450 million people globally [2,3]. HL is multifactorial and may have genetic and/or environmental etiology. Recent advances in genetic screening strategies have significantly improved our abilities to identify the variants underlying the disease etiology. For instance, whole exome sequencing (WES) plays a critical role in identifying causative DNA alterations in both common and rare Mendelian disorders, including HL [4,5]. A considerable number of human patients affected with HL have monogenic origin; however, many cases escape molecular identification [6]. An imperative explanation for such failure is inter- and intra-familial locus heterogeneity, where within a single family causal variants in two or more genes underlie disease etiology [7]. As example of intra- and inter-sibship familial locus heterogeneity, in this study, we present inheritance pattern of five rare variants in five known deafness genes with HL in a large consanguineous Pakistani family GCUFAHL38. Two of the affected individuals were homozygous for the variants in genes, *GJB2*, *MPDZ*, known to cause recessively inherited hearing loss, while other two affected individuals were heterozygous for a variant in *KCNQ4*, a dominant deafness-causing genes. Our study further highlights the complexity of inheritance mechanisms in hereditary HL and associated phenotypes in inbred population.

## 2. Materials and Methods

### 2.1. Ethics Statement and Clinical Evaluation

Family GCUFAHL38 was ascertained from Punjab province of Pakistan after an Institutional Review Board (IRB) committee approval from University of Maryland, USA and Government College University, Faisalabad, Pakistan. The current study followed ethical standards of Helsinki declaration. Written informed consents were obtained from all the participating individuals. Air and bone conduction pure tone audiometric examinations were performed on all the available individuals. Romberg and Tandem gait tests were performed to assess any vestibular dysfunction. Thyroid, lipid profile, blood coagulation and cardiac enzymes levels were evaluated in the serum samples from the available subjects. Visual acuity was also documented by an Ophthalmologist. Genomic DNA was isolated from the blood samples of the participants.

### 2.2. Whole Exome Sequencing and Bioinformatics Analyses

Whole exome sequencing was performed on the participants III:3, III:4, IV:2 and IV:3, using Agilent SureSelect Human Expanded All Exon kit and Illumina HiSeq4500 instrument with an average 100× coverage [8]. We filtered out variants present in non-coding regions, synonymous and read depth < 4 and included non-synonymous, splice site, frame shift and stop gained or retained with an allele frequency less than 0.001, predicted pathogenic variants by at least 2 programs, i.e., OMIM and Polyphen2 and CADD score > 12. Variants that pass our filtration criteria were Sanger sequenced in all the participating family members. Allele frequencies in the general population were assessed through gnomAD database (GnomAD: http://gnomad.broadinstitute.org; accessed on 3 March 2021). Pathogenicity predictions and the American College of Medical Genetics and Genomics (ACMG) classification of the identified genetic variants were assessed by online algorithm (GVIT: https://www.medschool.umaryland.edu/genetic_variant_interpretation_tool1.html/; accessed on 4 October 2021). Clustal omega was used to generate multiple sequence alignments of identified gene proteins (Clutal Omega: https://www.ebi.ac.uk/Tools/msa/clustalo/; accessed on 20 April 2021).

### 2.3. Structural Modeling

Phyre2 and chimera were used to generate and visualize protein 3D structures. Intensive mode option was used to generate 3D structures on Phyre2. Molprobity (MOLprobity: http://molprobity.biochem.duke.edu/index.php; accessed on 23 September 2021) was used to generate wild type (WT) and mutant proteins Ramachandran plots.

## 3. Results

### 3.1. Clinical Presentation

**Figure 1:**
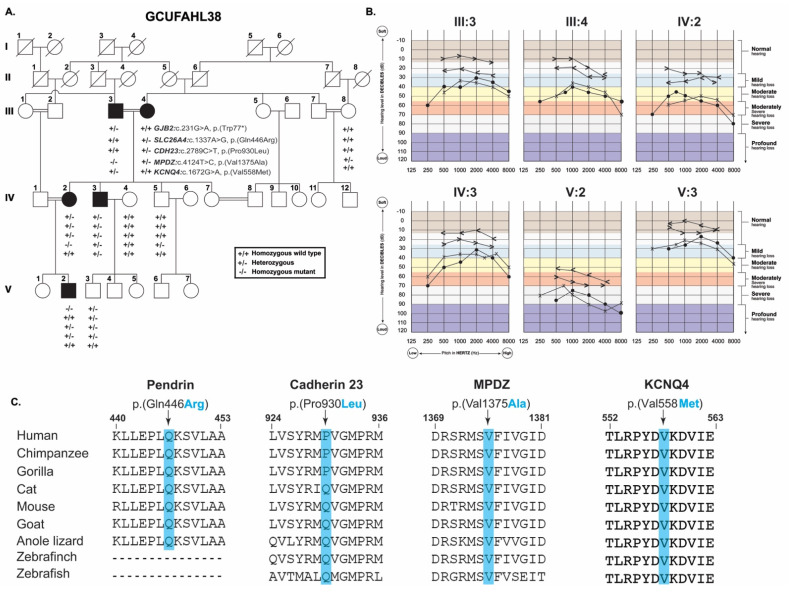
GCUFAHL38 family pedigree and audiometry. (**A**) Family pedigree affected with HL demonstrating inheritance pattern of five rare variants of five known deafness genes. Genotypes for each gene are shown in the form of haplotype under each individual checked for segregation analysis. Filled and empty circles represent affected and unaffected individuals, respectively; (**B**) Audiograms of affected individuals showing mild to severe–profound HL profile; (**C**) Multiple sequence alignment of hearing loss proteins amino acid residues found in study. Residues of interest are highlighted with blue bars.

The large consanguineous Pakistani family GCUFAHL38 comprised of five affected individuals in three generations impacted with post-lingual HL (Figure 1A). No audiometric data was available to confirm the HL onset; however, based on family history, age of disease onset was around 11 years, while in two of the affected individuals (III:4 and IV:2) it was further delayed. Pure-tone audiometric analyses in noise-free conditions (sound proof chamber was not available), revealed mild to severe–profound hearing loss in different individuals. Both consanguineous parents (III:3 and III:4) in third generation (Figure 1A) had bilateral mild to moderate and moderately severe HL, respectively (Figure 1B). While both of their affected children (IV:2 and IV:3) had moderate-to-severe HL, and their grandchild (V:2) had bilateral severe–profound HL (Figure 1B). Their cousin (V:3) had normal audiometric profile (Figure 1A,B). Detailed physical examination at the time of enrollment did not indicate any noticeable dysmorphic features among the affected individuals. Family reported no history of trauma, infections, or ototoxic medications. We did not find any significant vestibular deficits in all the affected individuals based on Romberg and Tandem gait tests. Ophthalmic examination did not reveal any apparent vision deficits, e.g., corneal opacity, central or peripheral visual field loss or night blindness in all the affected individuals (Table 1). Finally, blood biochemical parameters indicating thyroid, lipid metabolism, coagulation profile and cardiac enzymes were within normal range (Table 1).

### 3.2. Genetic Analysis

WES data of affected individuals III:3, III:4, IV:2, and IV:3 was filtered as described in the method section assuming both autosomal dominant as well as recessive inheritance pattern, and resulting candidate variants were Sanger sequenced in all the participating family members. Intriguingly, we found potentially pathogenic variants of five known HL genes, *GJB2*, *SLC26A4*, *CDH23*, *KCNQ4*, and *MPDZ*, in various combination of zygosity among the five affected individuals (Figure 1A). For instance, the proband (IV:2) was homozygous for a rare missense variant in *MPDZ:* c.4124T>C, p.(Val1375Ala), while heterozygous for *GJB2:* c.231G>A, p.(Trp77 *), *SLC26A4:* c.1337A>G, p.(Gln446Arg), and *CDH23:* c.2789C>T, p.(Pro930Leu) variants (Figure 1A). Other affected individuals inherited different combination of these five variants (Figure 1A). Individuals III:3 and IV:3 inherited a heterozygous novel missense variant, c.1672G>A, p.(Val558Met), of *KCNQ4*, a gene known to cause dominant deafness in humans [9].

Variants found in *GJB2* (c.231G>A), and *SLC26A4* (c.1337A>G) have been previously reported in families with HL [10,11,12], and have categorized as “pathogenic” or “likely-pathogenic” according to ACMG/AMP guidelines [13], while the other three variants are novel and based on the available evidences fall into the “uncertain significance” category [13,14] (Table 2). All the identified variants are predicted disease causing by multiple prediction programs (Table 2). The identified missense variants replaced relatively conserved residues in the encoded proteins (Figure 1C).

### 3.3. Protein Secondary Structure Modeling

**Figure 2:**
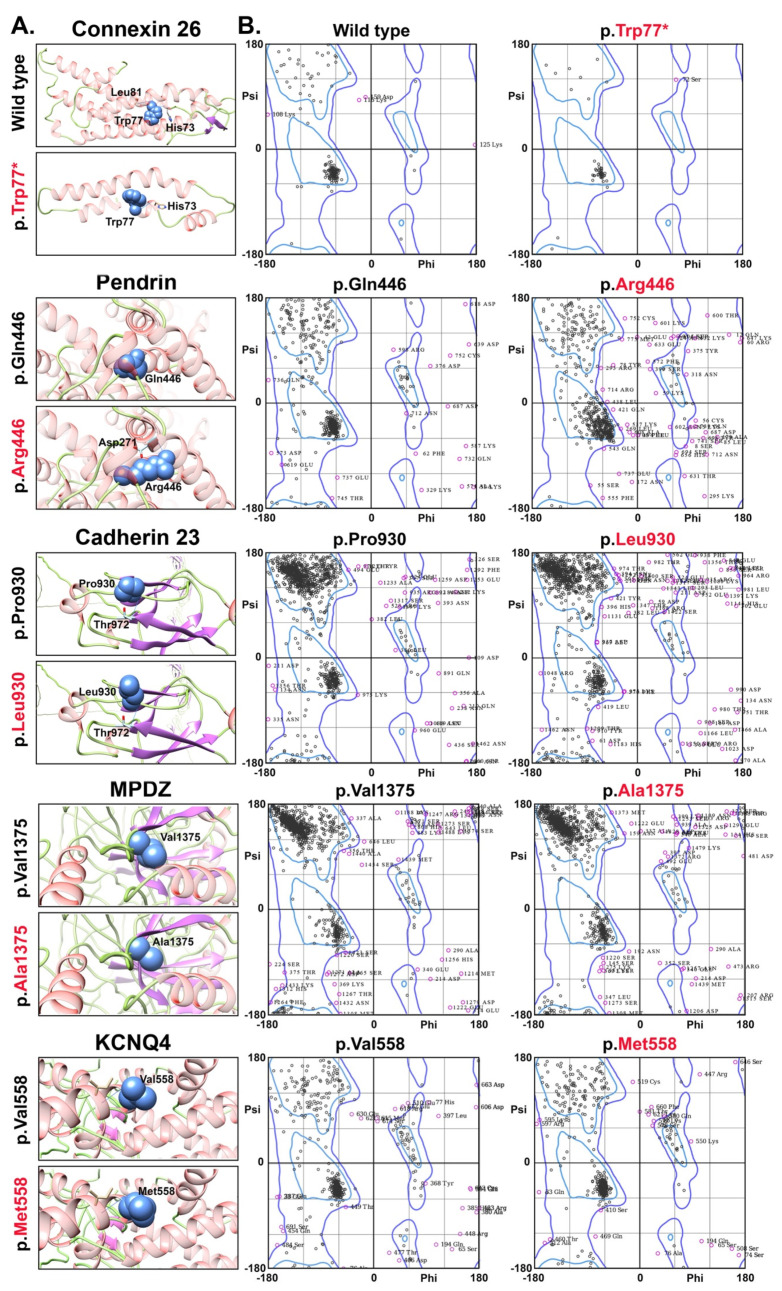
Hearing loss proteins 3D structures (close ups) and Ramachandran plots. (**A**) Protein secondary structure is labeled in following colors: Helix, pink; strand, purple and coils in green color. Concerned residues (shown in spheres) and hydrogen bonding pattern are represented in blue color. However, residues involved in hydrogen bonding with concerned residues are shown in stick form and colored by heteroatom. (**B**) Ramachandran plots are showing residues distribution for both wild type and mutant protein sequences side by side.

To understand the impact of identified variants, we used Phyre2 to generate protein’s 3D structures and HOPE prediction program [15]. *GJB2* variant p.(Trp77 *) results in early truncation of the encoded connexin 26 protein (Figure 2A), and may undergo nonsense mediated mRNA decay. The p.(Gln446Arg) variant of *SLC26A4,* encodes pendrin (a transmembrane exchanger of anions and bases), is predicted to introduce new hydrogen bonding pattern (Figure 2A) due to charge difference, and also impact the pendrin function due to position of the native residue within the sulfate transporter domain. Ramachandran plot analysis of wild type *SLC26A4* showed 89% residues to lie in favored region versus mutant protein (79%), similarly, 31 outlier residues in WT versus 73 in mutant (Figure 2B).

Cadherin 23, encoded by *CDH23*, missense variant p.(Pro930Leu) is located within the calcium binding extracellular cadherin repeat. The native proline residue is rigid in structure and may confine protein’s backbone into certain specific conformation and it’s substitution with leucine might disrupt the conformation as well as protein function (Figure 2A). Due to size limit, only first 1500 amino acids of cadherin 23 were used for the 3-D modeling and Ramachandran plots. Of these, 89% and 96% residues in WT protein were present in favored and allowed regions with 62 outliers, respectively. While the cadherin 23 harboring p.(Pro930Leu) substitution had 85% and 93% residues in favored and allowed regions with 98 outlier, respectively (Figure 2B). *KCNQ4* substitution p.(Val558Met) is located in the potassium channel domain, important to facilitate energy-independent K+ diffusion through transmembrane aqueous channels. As per molecular prediction, mutant residue methionine is bigger in size than wild type residue and as p.Val558 is located on the surface of the protein, its substitution leads towards the distorted interactions with other molecules. Ramachandran plot results showed 52 outliers in WT *KCNQ4* protein versus mutant (40). In WT protein, 82% and 92% residues were found in favored and allowed regions, respectively, versus mutant protein (84% and 94%) (Figure 2B).

The missense variant p.(Val1375Ala) is located in the predicted PDZ domain of the encoded *MPDZ* (multiple PDZ domain) protein. PDZ domains are comprised of 80–90 amino acids (six β-strands and two α-helices), which are compactly arranged to form globular structures. The p.(Val1375Ala) substitution is predicted to result in loss of interaction and disturb protein’s binding activity due to smaller size of alanine residue (Figure 2B). However, we did not find any significant difference in Ramachandran plot analysis of both WT and mutant *MPDZ* proteins (Figure 2B).

## 4. Discussion

Hearing loss is highly genetically heterogeneous disease, and one of the significant challenges of uncovering the underlying pathogenic variants is inter- and intra-familial locus heterogeneity [7]. This study presents a HL family GCUFAHL38, in which five different deafness genes (*GJB2*, *SLC26A4*, *CDH23*, *MPDZ*, *KCNQ4*) rare variants were co-segregating in different configurations (Figure 1A), and thus further highlights the genetic complexity of hearing disorders in highly inbred families. Despite the exome sequencing of four family members, we were only able to identify definitive causal variant in one affected individual, V:2, that had homozygous c.231G>A (p.(Trp77 *)) allele of *GJB2*. In three (III:3, IV:2, and IV:3) of the remaining four affected individuals (Figure 1A), although we found rare and predicted damaging variants of known deafness genes (*MPDZ*, *KCNQ4*), however, these variants require further experimental studies to validate their pathogenic impact on the encoded proteins. Thus, our study also underscores the necessity of functional studies together with comprehensive genetic profiling to reach a conclusive genetic diagnosis in highly inbred families.

In humans, pathogenic variants of *GJB2* (gap junction protein β) have been widely reported to cause autosomal recessive HL. Connexin-26 gap junction protein encoded by *GJB2*, plays a vital role in homeostasis of cochlear fluids [16]. The p.(Trp77 *) nonsense variant, found in homozygous fashion in individual V:2, is a well-documented HL-causing allele of *GJB2* [10]. Similarly, the p.(Gln446Arg) variant of *SLC26A4* (solute carrier family 26, member 4) has been associated with HL in previous studies. However, the variants of *CDH23*, *MPDZ* and *KCNQ4* are novel. Our 3D protein topological analysis predicted deleterious impact of all the novel variants identified here, however, further studies including evaluation in appropriate animal models would be required to establish their pathological effect on the inner ear development and hearing function. *GJB2* and *SLC26A4* both are considered among common HL genes in Pakistani population [17]. Similarly, genetic variants in *CDH23* are also commonly found in cases of Usher syndrome, a deaf–blindness disorder [18].

Although associated with HL in mice and human [19,20], our study represents the second citation of two deaf individuals homozygous for *MPDZ* variant. *MPDZ*, mapped on human chromosome 9p23, encodes PDZ domains containing protein. A previous study reported three human families affected with congenital hydrocephalus, harboring *MPDZ* variants [Family 1, p.(Gln1490Argfs*19); Family 2, p.(Arg744*); p.(Arg1071*); Family 3, p.(Ala1760Thr)] [21]. Along with hydrocephalus, other clinical features of the patients include delayed motor development, dysmorphic facial features, foveal dysplasia, iris coloboma, atrial septal defect (ASD) lung hypoplasia, hypotonia, portal vein thrombosis or sensorineural HL. In contrast, individuals (III:3 and IV:2) of family GCUFAHL38 homozygous for p.(Val1375Ala) variant of *MPDZ* had only deafness. Furthermore, a recent study reported two affected siblings with non-syndromic hearing sensorineural HL associated with p.(Pro379His) variant in *MPDZ* [19]. Although the current sample size is not large enough, taken together, these studies might indicate a possible genotype–phenotype correlation, in which the missense variants, plausibly hypomorphic alleles, cause HL while truncating variants of *MPDZ* causing multiple organs syndrome.

Among the five members of KCNQ family of voltage-gated potassium channels, variants in *KCNQ4* are reported to cause autosomal dominant non-syndromic hearing loss in humans [9]. Furthermore, several single nucleotide polymorphisms in *KCNQ4* are also reported to contribute towards age-related hearing loss, which is a complex disease rising from an alliance between genetic and environmental facets [22,23]. *KCNQ4* is known to play a crucial role in the electromobility of cochlear outer hair cells [24], as well as synchronizing K+ recycling and homeostasis in the inner ear [25]. As of October 2021, around 72 mutations in *KCNQ4* have been reported to cause HL in humans, with a potential genotype–phenotype correlation, in which the truncating variants have been associated with severe phenotype as compared to non-truncating variants of *KCNQ4* [26]. Both affected individuals of family GCUFAHL38 that inherited p. (Val558Met) variant of *KCNQ4* had non-syndromic mild to moderate HL (Figure 1B).

In summary, we showed an evidence of HL human family displaying an extreme genetic heterogeneity with co-occurrence of multiple rare variants of known disease-causing genes. Among all these rare variants, only two of them {c.231G>A; p.(Trp77 *) allele of *GJB2* and c.1377A>G; p.(Gln446Arg) of *SLC26A4*} have been previously classified as “pathogenic” or “likely pathogenic”, respectively, based on ACMG/AMP guidelines, while the remaining three novel variants found in this study require further genetics and functional support to conclusively determine their causality. In order to find the genetic basis of complex disorders, sometimes simple linkage analysis and homozygosity mapping may not work as even sibs have different genes rare variants [7]. In silico predictions, bioinformatics analyses, and even pooling of multiple affected DNA samples and exome may also not work. In our opinion, comprehensive phenotyping, individualized exome sequencing coupled with the established functional assays could be a solution for complete genetic diagnosis.

## Figures and Tables

**Table 1:** Clinical evaluation of four members of GCUFAHL38 family.

Subject		III:3	III:4	IV:3	IV:2
Gender		M	F	M	F
Age (years)		86	85	45	50
Ethnicity		Punjab			
Status		Affected	Affected	Affected	Affected
Comorbidity		Nil	Nil	Nil	Nil
Hearing loss		Yes	Yes	Yes	Yes
Thyroid profile		Normal	Normal	Normal	Normal
Lipid profile		Normal	Normal	Normal	Normal
Blood coagulation profile		Normal	Normal	Normal	Normal
Cardiac enzymes profile		Normal	Normal	Normal	Normal
Visual acuity	Right	6/8	6/8	6/6	6/6
	Left	6/9	6/6	6/6	6/6

**Table 2:** Genes, identified variants and their ACMG classification in GCUFAHL38.

Family	GCUFAHL38				
Gene	*GJB2*	*SLC26A4*	*CDH23*	*MPDZ*	*KCNQ4*
Transcript ID	NM_004004.6	NM_000441.2	NM_022124.6	NM_001378778	NM_004700.4
cDNA change	c.231G>A	c.1337A>G	c.2789C>T	c.4124T>C	c.1672G>A
Amino acid change	p.(Trp77 *)	p.(Gln446Arg)	p.(Pro930Leu)	p.(Val1375Ala)	p.(Val558Met)
gnomAD	0.0001392	0.00007578	0.00006791	0.00002410	0.00001193
ACMG (criteria used)	M1: Pathogenic (PVS1, PP5, PM2, PP3)	M2: Likely pathogenic (PS1, PS4, PM2, PM3, PP3)	M3: Unknown significance (PM1, PM2, BP4)	M3: Unknown significance (PP1, PM2, PM3, PP3, BP1)	M3: Unknown significance (PM1, PM2, PP3)
CADD	39	26	21	33	28
DANN	0.99	0.99	0.94	0.99	0.99
MutationTaster	Disease causing	Disease causing	Disease causing	Disease causing	Disease causing
FATHMM-MKL	Damaging	Damaging	Damaging	Damaging	Damaging
LRT	Deleterious	Deleterious	Deleterious	Deleterious	Deleterious
EIGEN PC	Pathogenic	Pathogenic	Benign	Pathogenic	Pathogenic
SIFT	NA	Tolerated	Tolerated	Damaging	Damaging
MutPred	NA	Pathogenic	Pathogenic	Pathogenic	Pathogenic
Provean	NA	Damaging	NA	Damaging	Damaging
GERP	5.32	5.92	5.5	5.71	5.11

PVS1: Null variant (nonsense, frameshift, canonical ±1 or 2 splice sites, initiation codon, single or multiexon deletion) in a gene where LOF is a known mechanism of disease. PS1: Same amino acid change as a previously established pathogenic variant regardless of nucleotide change. PS4: The prevalence of the variant in affected individuals is significantly increased compared with the prevalence in controls. PP1: Cosegregation with disease in multiple affected family members in a gene definitively known to cause the disease. PM1: Located in a mutational hot spot and/or critical and well-established functional domain (e.g., active site of an enzyme) without benign variation. PM2 Absent from controls (or at extremely low frequency if recessive) in Exome Sequencing Project, 1000 Genomes Project, or Exome Aggregation Consortium. PP3 Multiple lines of computational evidence support a deleterious effect on the gene or gene product (conservation, evolutionary, splicing impact, etc.). BP1 Missense variant in a gene for which primarily truncating variants are known to cause disease. BP4 Multiple lines of computational evidence suggest no impact on gene or gene product (conservation, evolutionary, splicing impact, etc.).

## Data Availability

Not applicable.
